# Flash glucose monitoring in gestational diabetes mellitus (FLAMINGO): a randomised controlled trial

**DOI:** 10.1007/s00592-023-02091-2

**Published:** 2023-05-10

**Authors:** Agata Majewska, Paweł Jan Stanirowski, Jacek Tatur, Barbara Wojda, Iwona Radosz, Mirosław Wielgos, Dorota Agata Bomba-Opon

**Affiliations:** 1 1st Department of Obstetrics and Gynaecology, Medical University of Warsaw, Starynkiewicza Square 1/3, 02-015 Warsaw, Poland; 2 Polish Society of Gynecologists and Obstetricians, Club 35, 02-677 Warsaw, Poland; 3 Department of Nutrition and Nutritional Value of Food, National Institute of Public Health NIH-National Research Institute, Chocimska St. 24, 00-791 Warsaw, Poland; 4 Lazarski University, Warsaw, Poland; 5 Collegium Medicum Jan Kochanowski University of Kielce, Kielce, Poland

**Keywords:** Flash glucose monitoring, Gestational diabetes mellitus, Dysglycaemia, Macrosomia

## Abstract

**Aims:**

Gestational diabetes mellitus (GDM) is the most common type of hyperglycaemia in pregnancy. GDM is a risk factor of adverse perinatal outcomes, with the incidence rate increasing proportionally to the level of maternal dysglycaemia. Therefore, glycaemic control plays an important role in management of GDM. The aim of this study was to assess the efficacy of flash glucose monitoring (FGM) in GDM.

**Materials and methods:**

This was a non-blinded, randomised controlled trial, that recruited 100 pregnant women diagnosed with GDM between 24 and 28 weeks of gestation at the 1st Department of Obstetrics and Gynaecology, Medical University of Warsaw. After meeting the inclusion criteria patients were randomly allocated to the study group (FGM, *n *= 50) or control group (self-monitoring of blood glucose—SMBG, *n* = 50). Clinical and laboratory results were assessed at four follow-up visits. The primary outcome was mean fasting and postprandial glycaemia. The secondary outcomes were perinatal outcomes.

**Results:**

There was no significant difference in mean glycaemia between the groups (*p* = 0.437) Compared to the control group, the study group significantly reduced their fasting (*p* = 0.027) and postprandial glycaemia (*p* = 0.034) during the first 4 weeks following GDM diagnosis, with no significant difference in progression to insulin therapy (OR 1.09, 95% CI 0.47–2.57)**.** Incidence of fetal macrosomia was significantly higher in SMBG as compared to FGM group (OR 5.63, 95% CI 1.16–27.22).

**Conclusions:**

Study results indicate that FGM has an impact on glycaemic control, dietary habits and incidence of fetal macrosomia in patients with GDM.

*Trial registration clinicaltrials.gov ID*: NCT04422821.

**Supplementary Information:**

The online version contains supplementary material available at 10.1007/s00592-023-02091-2.

## Introduction

Gestational diabetes mellitus (GDM) is the most common type of hyperglycaemia in pregnancy, with the incidence rate of 14% varying worldwide [1]. GDM is a risk factor of adverse perinatal outcomes, including fetal macrosomia, shoulder dystocia and neonatal hypoglycaemia [2, 3]. It is believed, that adverse perinatal outcomes correlate proportionally to the level of maternal dysglycaemia [2, 4, 5]. Therefore, glycaemic control is suggested to play an important role in management of GDM [6]. There are various options of glycaemic control with self-monitoring of blood glucose (SMBG) being the most common method and a standard of care for pregnant women [7]. In recent years, however, new methods have been introduced, namely continuous glucose monitoring systems (CGM), which include real-time continuous glucose monitoring (rtCGM), and flash glucose monitoring (FGM) also known as intermittently scanned continuous glucose monitoring (isCGM) systems [8]. Superiority of both is flexibility and accessibility to circadian glycaemia due to constant glycaemia measurements, that might lead to better patient’s compliance [9, 10]. Additionally, rtCGM as well as FGM do not require finger pricking for each glycaemia assessment, and consequently improve the quality of life of diabetic patients [11]. Currently, both systems are commonly used in pre-gestational diabetes mellitus [11, 12]; however, increasing number of data suggest that they could be of benefit for glucose monitoring in GDM-complicated pregnancies [13]. In previous studies it has been shown, that rtCGM improved glycaemic control, had an impact on lifestyle changes, such as diet modifications and led to better qualification to insulin therapy in GDM women [14–16]. Nevertheless, it is not clear, whether these systems have an impact on perinatal outcomes, such as incidence of fetal macrosomia or neonatal hypoglycaemia in this population. Therefore, in the present study we aimed to compare FGM with SMBG and to analyse the efficacy of the both methods in GDM management.

## Materials and methods

We performed a non-blinded, randomised controlled trial between March 2020 and October 2022, that recruited 100 pregnant women diagnosed with GDM between 24 and 28 weeks of gestation at the 1st Department of Obstetrics and Gynaecology, Medical University of Warsaw. A diagnosis of GDM was based on 2013 World Health Organisation criteria: (1) fasting plasma glucose 92–125 mg/dl (2) 1-h glucose concentration ≥ 180 mg/dl or (3) 2-h glucose concentration 153–199 mg/dl [17]. The inclusion criteria for the study were: patient’s age > 18 years old and singleton pregnancy. The exclusion criteria included: multiple pregnancy, fetal malformations, pre-gestational diabetes mellitus, chronic or pregnancy-induced hypertension, chronic renal or hepatic disease diagnosed prior to study entry, in vitro fertilisation, premature rupture of membranes, placenta praevia, smoking in pregnancy, as well as intake of medications including: methyldopa, tetracyclin, acetylosalicylic acid, acetaminofen, ibuprofen, L-dopa, tolazamide or tolbutamide.

After meeting the inclusion criteria patients were randomly allocated with a nested qualitative evaluation and 1:1 allocation ratio to the study (*n* = 50) or control group (*n* = 50). Simple randomisation with the computer-generated list was used for patient’s randomisation process by the non-member of the trial research staff. During the first 4 weeks following the diagnosis of GDM participants in the study group measured glucose concentrations using the FGM system (Freestyle Libre 1, Abbott Diabetes Care, Alameda, California, USA), whereas the control group used the SMBG (iXell; Genexo sp; Warsaw, Poland; ISO 15197:2015). In the following weeks until birth, glucose concentrations were measured exclusively with SMBG method in both groups.

Study consisted of five visits, including recruitment and four follow-up visits. At the first visit all study participants were informed about glycaemic control, diet recommendations and physical activity. During the three follow up visits (2 weeks, 4 weeks after the recruitment visit and between 34–36 weeks of gestation) clinical and laboratory results were assessed, including: fasting and 1 h-postprandial glucose concentrations (after breakfast, lunch, and dinner), qualification to insulin therapy and dosage, diet control, physical activity and gestational weight gain. The qualification to insulin therapy was decided in case of hyperglycaemia, defined as fasting glycaemia ≥ 90 mg/dl or 1 h-postprandial glycaemia ≥ 140 mg/dl. At the second and third follow up visits Haemoglobin A1c (HbA1c) concentration and ultrasound estimated fetal weight (EFW) were measured. For the assessment of fetal birthweight percentile INTERGROWTH-21st Chart was implemented. At the last follow up visit (Visit 5; postpartum) patients were reviewed for perinatal outcomes: weeks of gestation and route of birth, newborn weight and neonatal hypoglycaemic events.

As part of our study we analysed physical activity by daily footsteps measure with step counter app. After calculating daily steps, we divided participants into 4 groups–sedentary: < 5000; low active: 5000–7500; somewhat active: 7500–10,000 or active: > 10,000 steps.

We also evaluated patient’s dietary habits and its modifications throughout pregnancy by using Eating Assessment Test (EAT) prepared by the Polish National Institute of Public Health–National Institute of Hygiene (Supplementary File 1). EAT contained a short questionnaire, with 20 items for diet assessment. Based on EAT, participants were assigned to one of four diet groups: good: 39–42; satisfactory: 30–38; demanding diet modification: 12–29 and not satisfactory: < 12 points.

The primary outcome of the study was mean fasting and 1 h postprandial glycaemia during the first 28 days following GDM diagnosis. Additionally, we analysed variability pattern of glucose concentration inside the groups by using delta mean fasting and postprandial glucose concentration for each group, defined as difference between 3rd to 4th week mean glycaemia and 1st to 2nd week mean glycaemia. The secondary outcomes were maternal and neonatal outcomes, including: qualification to insulin therapy, insulin dosage, hypoglycaemic episodes (defined as glycaemia < 70 mg/dl), HbA1c concentration, pregnancy weight gain, physical activity and diet modifications, fetal birthweight and percentile, incidence of fetal macrosomia and neonatal hypoglycaemia.

The detailed study protocol was already published in 2020 [18]. The study protocol was approved by Ethics Committee at the Medical University of Warsaw (KB/50/2020) and trial was registered in ClinicalTrials.gov (registration number: NCT04422821). All women eligible for the study provided written informed consent prior to enrolment.

### Sample size calculation

We performed a two-sided power analysis (power of 80%, significance level of 5%) and estimated a sample size of 80 patients (40 patients for each group). The sample size was further increased to 100 patients based on estimated drop-out rate of 10% participants. For the estimation, we used results of a previous study comparing target glycaemic range between FGM and SMBG [19].

### Statistical analysis

The statistical analysis was carried out using STATA, version 17.0 (Stata Corporation, TX, USA) and GraphPad Prism v9 (GraphPad Software, CA, USA). Continuous variables were presented as means ± standard deviation (SD) or medians ± interquartile range (IQR) and categorical variables were presented as frequencies (%) by treatment group. Normal distribution of continuous variables was assessed using the Shapiro–Wilk’s test. For the analysis of continuous variables either Mann–Whitney U test or t-test were used and for categorical variables Fisher exact test was applied. For the correlation analysis, we used Pearson correlation coefficient. To estimate correlation between method of glycaemic control and perinatal outcomes we performed binary logistic regression and presented results as odds ratio (OR) and 95% confidence interval (95% CI). We performed linear regression to analyse relationship between glucose concentration and continuous perinatal outcomes. All statistical tests applied were two-sided. A *p*-value of less than 0.05 was a cut-off for significant difference.

## Results

Between March 2020 and October 2022, we recruited 100 patients that met the inclusion criteria. 50 patients were recruited to the study group, out of which 49 completed the study (one patient was excluded after recruitment process at the first follow up visit due to diagnosis of placenta praevia). 50 patients were allocated to the control group, with 50 women included in the statistical analysis. Maternal baseline characteristics did not differ significantly between the groups (Table 1).

**Table 1:** Maternal baseline demographic and clinical characteristics

	Study group (*n* = 49)	Control group (*n* = 50)	*p*-value
Maternal age (age), median (IQR)	33 (31–37)	32 (28–34)	n/s*** 0.08
BMI* before pregnancy (kg/m^2^), median (IQR)	24.65 (21.65–27.24)	22.95 (20.80–26.03)	n/s 0.96
Multiparous,* n* (%)	26 (53%)	25 (50%)	n/s 0.31
Weeks of gestation at the recruitment visit (weeks), median (IQR)	27 (26–28)	27 (26–28)	n/s 0.81
OGTT**, fasting glycaemia (mg/dl), median (IQR)	87 (79–96.4)	92 (85–94)	n/s 0.47
1 h OGTT (mg/dl), median (IQR)	186 (156–196)	181.5 (149–189)	n/s 0.62
2 h OGTT (mg/dl), median (IQR)	143 (121–161)	138.5 (112–158)	n/s 0.70

*BMI- Body mass index; **OGTT–Oral glucose tolerance test; *** not significant

### Primary outcome

There was no significant difference in mean (SD) fasting glucose concentration during the first 4 weeks following GDM diagnosis between the groups (*p* = 0.437), whereas the mean postprandial glycaemia differed significantly, with lower concentration in the control group (*p* = 0.011) (Table 2).

**Table 2:** Glycaemic control in the first 4 weeks following inclusion to the study

	Study group (*n* = 49)	Control group (*n* = 50)	*p*-value
Mean fasting glycaemia during the first month (mg/dl), mean (SD)	86.71 (7.59)	85.10 (7.37)	*p* = 0.437
Mean postprandial glycaemia during the first month (mg/dl), mean (SD)	113.94 (8.13)	109.52 (6.36)	*p* = 0.011
Delta mean fasting glycaemia (mg/dl), mean (SD)	−0.69 (7.89)	2.52 (3.53)	*p* = 0.027
Delta mean postprandial glycaemia (mg/dl), mean (SD)	−1.01 (7.62)	1.93 (4.79)	*p* = 0.033

**Fig. 1:**
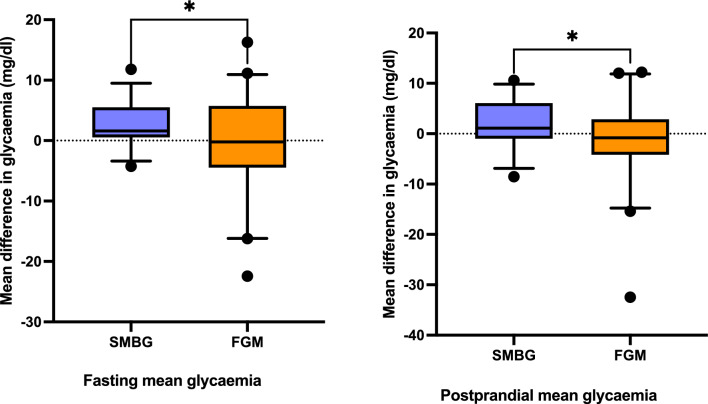
Mean difference in glycaemia between 14–28 and 1–13 days following GDM diagnosis On the left: Mean difference in fasting glycaemia between 14–28 and 1–13 days following GDM diagnosis. On the right: Mean difference in postprandial glycaemia between 14–28 and 1–13 days following GDM diagnosis. Abbreviations: GDM: gestational diabetes mellitus. SMBG: self-monitoring of blood glucose. FGM: flash glucose monitoring

Delta mean glucose concentrations were significantly reduced in the study group in comparison to the control group, with lower delta fasting (−0.69 mg/dl (7.89) vs. 2.52 mg/dl (3.53; *p* = 0.027)) and delta postprandial mean glycaemia (−1.01 mg/dl (7.62) vs. 1.93 mg/dl (4.79; *p* = 0.034)) in the FGM group in the third and fourth week following the inclusion to the study (Fig. 1).

### Secondary outcomes

FGM enabled to reveal all nocturnal hypoglycaemic events during the first month of the study in the study group (mean incidence of 15 events/month). In 6 out of 49 patients (12.24%) we recorded one episode of hypoglycaemic event level 2 (glucose concentration less than 54 mg/dl). We were not able to compare it with the control group, as participants from the control group measured glucose concentration only once between 2:00 and 4:00 at night. Therefore, in the control group the mean incidence of nocturnal hypoglycaemic events was 2 events per month, but the result was not comparable with the FGM group.

We analysed HbA1c concentration at the 1st, 3rd and 4th visit and found no significant difference between the groups (Supplementary Appendix 1). Delta HbA1c concentration, defined as difference between HbA1c measured between 34–36 weeks of gestation and HbA1c measured at the recruitment visit also did not differ significantly between the groups (ΔHbA1c for SMBG group was 0.1% (−0.1–0.2) and for FGM group 0.05% (−0.2–0.2), *p*-value 0.546). There were also no significant differences between the groups regarding qualification to insulin therapy (32% from the control group vs. 30.61% from the study group; OR 1.09, 95% CI 0.47–2.57). Long-acting insulin dosage at the follow up visit between 34–36 weeks of gestation did not differ between the groups (*p* = 0.199) (Supplementary Appendix 1). One participant from the control group (2%), whereas three participants from the study group (6.12%) demanded short-acting insulin therapy.

We found no significant difference in incidence of caesarean section (OR 0.84, 95% CI 038–1.87) and weeks of gestation at birth between the groups (*p* = 0.872). There was significantly higher incidence of fetal macrosomia ≥ 4000 g in the SMBG group (20% vs. 4.08%, OR 5.62, 95% CI 1.16−27.22); large for gestational age neonates (defined as birthweight > 90 percentile) and neonatal hypoglycaemia also appeared more often in the control group, but the differences were not statistically significant (OR 2.38, 95% CI 0.69–8.22, and OR 1.29, 95% CI 0.50–3.28 respectively). Median birthweight percentiles (INTERGROWTH-21st standards) did not differ significantly between the groups (*p* = 0.697).

**Fig. 2:**
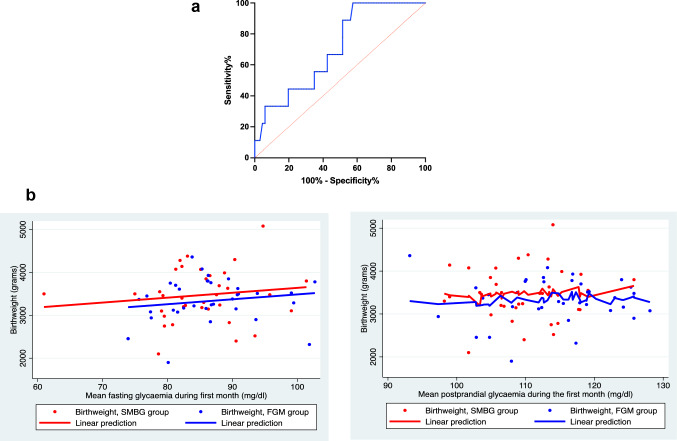
Relationship between glycaemia during the first month of the study and birthweight **A** ROC curve for association between postprandial glucose concentration and fetal macrosomia. **B** Linear regression for relationship between: fasting glycaemia during the first month of the study and birthweight (on the left), and postprandial glycaemia during the first month of the study and birthweight (on the right). Abbreviations: ROC: receiver operating characteristic curve

Logistic regression did not reveal strong association between mean glycaemia during the first month and incidence of neonatal hypoglycaemia, with OR 0.96 (95% CI 0.88–1.05) for fasting mean glycaemia and OR 0.99 (95% CI 0.91–1.08) for postprandial mean glycaemia, respectively. However, postprandial glucose concentration was correlated with the incidence of fetal macrosomia (ROC AUC 0.704, 95% CI 0.546–0.862) (Fig. 2A).

We used Pearson’s correlation to analyse, whether mean glucose concentration during the first month of the study was correlated with birthweight, but we found no strong relationship between either fasting mean glycaemia (*r* = 0.118, *p* = 0.307) or postprandial mean glycaemia (*r* = 0−0.010, *p* = 0.931) and newborn weight. A linear regression established, that both fasting and postprandial mean glycaemia could not significantly predict the fetal birthweight (*F* (1, 75) = 0.01, *p* = 0.307 and * F*(1, 75) = 0.01, *p* = 0.93, respectively) (Fig. 2B).

Gestational weight gain, defined as difference between weight at the 3rd follow up visit and weight at the recruitment visit, did not differ significantly between the groups (*p* = 0.682) (Supplementary Appendix 1). We also analysed physical activity based on daily steps and allocation to the four groups. At the follow up visit between 34–36 weeks of gestation 22 participants from the control group (44%) and 16 participants from the study group (32.65%) were allocated to the sedentary group (< 5000 steps/day), whereas 13 patients from the SMBG group and 7 from the FGM group to the somewhat active or active group (at least 7500steps/day). There was no significant difference in allocation to the physical activity groups between the study and the control group (*p* = 0.302).

The study group more frequently modified their diet habits, when compared to the control group. Median (IQR) EAT score did not differ at the recruitment visit (34 points (28–36) for FGM compared to 33 points (28–35) for SMBG, *p* = 0.372), but between 34 and 36 weeks of gestation was significantly higher in the study group (37 points (34–39) vs. 34 points (33–37), *p* = 0.017). In FGM group we collected additional data about time in range (TIR) (marker showing glycaemic variability), with the median (IQR) of 96% (92–98%).

## Discussion

Our study revealed, that FGM might improve glycaemic control, when compared to SMBG. Although mean glycaemia did not differ significantly between the groups, and when separately analysed, postprandial glycaemia was significantly lower in the control group, glycaemia through the first month of the study improved more significantly in the study group (delta mean glucose concentration showed a pattern of decreasing changes in glycaemia in FGM group). As in previous studies on pre-gestational diabetes population, we found that FGM system led to strict glycaemic control, that consequently decreased mean fasting and postprandial glycaemias at follow up visits.

FLAMINGO revealed, that FGM had an impact on decreasing incidence of fetal macrosomia (birthweight > 4000 g) in GDM patients. It is consistent with the randomised controlled trial (RCT) by Murphy et al., evaluating effectiveness of CGM in pregnant women with type 1 and 2 diabetes [11]. LGA incidence was also lower in FGM group; however, the result was not statistically significant. In our RCT we found no significant difference in birthweight percentile between the groups. This outcome disagrees with the recent meta-analysis about CGM in pregnancy complicated with GDM, that found significantly lower birthweight in CGM group, compared to SMBG group [20]. Furthermore, this is contradictory outcome to the FLAMINGO result of lower macrosomia incidence in FGM group; the potential reason might be wide upper and lower limit for birthweight in both groups, that led to heterogeneous birth data included in our study and therefore non-significantly lower birthweight and percentile in the FGM group.

The CONCEPTT trial provided evidence that CGM led to clinically significant reduction in neonatal hypoglycaemia and NICU (neonatal intensive care unit) admission incidence [12]. However, we found no significant difference between FGM and SMBG group in incidence of hypoglycaemic event in newborns. As none of our patients was admitted to NICU, we could not compare it with available studies.

We found higher detection rate of hypoglycaemia in FGM group; however, these hypoglycaemic events were in most cases qualified as mild and none of them were symptomatic. As presented in previous studies continuous glucose monitoring detects masked hypoglycaemic events in pregnancy, that if qualified as mild, are clinically non-significant[21].

Interestingly, we found, that the study group was more prone to modify their diet habits, compared to the control group; however, it had no impact on gestational weight gain and qualification to insulin therapy. Our data differ from the previous studies, in which these outcomes were improved, when CGM systems were used [22, 23].

Our study has several strengths. To our knowledge, this is the first study with such a long assessment of glycaemia with FGM system in gestational diabetes mellitus. The data were derived from a randomised controlled trial, that diminishes the risk of bias that might be the consequence of the recruitment process. Furthermore, the baseline characteristics of the participants did not differ significantly between the groups and therefore adjustments had no significant impact on the analysed outcomes. All the additional results, including diet modifications, physical activity, were assessed using standardised tools. For the control group, we used one type of glucose meters to diminish the influence of different types of devices on primary outcome [24].

We also acknowledge some limitations. In FLAMINGO trial, the analysed time-period of glycaemic control was only 4 weeks, and therefore did not include glycaemic fluctuations occurring after the first month from GDM diagnosis till birth. Additionally, the EAT questionnaire was filled in by the patients and the diet scheme was not unified for all participants, that might produce the risk of bias.

## Conclusions

In summary, FGM application resulted in significantly better improvement in glycaemic control in the 3rd and 4th week of the study. FGM led to higher EAT score, that might indicate better diet modifications after GDM diagnosis; however, it had no impact on lifestyle interventions including gestational weight gain, qualification to insulin therapy or dosage of insulin. FGM significantly decreased incidence of fetal macrosomia, but had no significant impact on birthweight percentile or neonatal hypoglycaemia incidence. Therefore, further studies are needed to analyse the impact of FGM on improving perinatal outcomes in GDM-complicated pregnancies.

## Supplementary Information

Below is the link to the electronic supplementary material.

Supplementary file1 (DOCX 95 KB)
