# Correction: Flash glucose monitoring in gestational diabetes mellitus (FLAMINGO): a randomised controlled trial

**DOI:** 10.1007/s00592-023-02159-z

**Published:** 2023-08-10

**Authors:** Agata Majewska, Paweł Jan Stanirowski, Jacek Tatur, Barbara Wojda, Iwona Radosz, Mirosław Wielgos, Dorota Agata Bomba‑Opon

**Affiliations:** 1 1st Department of Obstetrics and Gynaecology, Medical University of Warsaw, Starynkiewicza Square 1/3, 02‑015 Warsaw, Poland; 2 Polish Society of Gynecologists and Obstetricians, Club 35, 02‑677 Warsaw, Poland; 3 Department of Nutrition and Nutritional Value of Food, National Institute of Public Health NIH-National Research Institute, Chocimska St. 24, 00‑791 Warsaw, Poland; 4 Lazarski University, Warsaw, Poland; 5 Collegium Medicum Jan Kochanowski University of Kielce, Kielce, Poland

## Correction to: Acta Diabetologica **https://doi.org/10.1007/s00592-023-02091-2**

Author would like to correct below error to their publication.

In Materials and methods section, first paragraph line 9 should read as “……..concentration 153–199 mg/dl [17].”

The original article has been corrected.

