# Supplementary material for: Flash glucose monitoring in gestational diabetes mellitus (FLAMINGO): a randomised controlled trial
Source: Acta Diabetol. 2023 May 10;60(9):1171–7. doi: 10.1007/s00592-023-02091-2 (PMC10359198; doi:10.1007/s00592-023-02091-2)
Supplement: Supplementary file 1 — Supplementary file1 (DOCX 95 KB) [file 592_2023_2091_MOESM1_ESM.docx]

**Supplementary Appendix 1. Maternal and neonatal outcomes**

|  | **Study group (n=49)** | **Control group (n=50)** | **p-value** |
| --- | --- | --- | --- |
| **HbA1c (%), Visit 1, median (IQR)**  **HbA1c (mmol/mol), Visit 1, median (IQR)** | 4.9 (4.6 – 5.2) 30 (27 – 32) | 4.9 (4.6 – 5.2) 30 (27 – 32) | p=0.782 |
| **HbA1c (%), Visit 3, median (IQR) HbA1c (mmol/mol), Visit 3, median (IQR)** | 5.1 (4.7 – 5.4) 32 (28 – 36) | 5 (4.8 – 5.2) 31 (29 – 32) | p=0.409 |
| **HbA1c (%), Visit 4, median (IQR) HbA1c (mmol/mol), Visit 4, median (IQR)** | 5.1 (4.8 – 5.4) 32 (29 – 36) | 5.1 (4.9 – 5.3) 32 (30 -34) | p=0.802 |
| **Insulin therapy, n (%)**   - **Long-acting, n (%)** - **Short-acting, n (%)** | 15 (30.61%)  15 (30.61%) 3 (6.12%) | 16 (32%)  16 (32%)  1 (2%) | p=0.827 |
| **Dosage of long-acting insulin at the third follow up visit, median (IQR)** | 8 (4 - 18) | 16 (8 – 20) | p=0.199 |
| **Gestational weight gain (kg), median (IQR)** | 2 (1-4) | 3 (1-4) | p=0.682 |
| **Weeks of gestation at birth  (weeks ^+ days^), median (IQR)** | 39^+4^ (38^+2^ – 39^+5^) | 39^+5^ (38^+3^ – 39^+5^) | p=0.872 |
| **Caesarean section, n (%)** | 25 (51.02%) | 25 (50%) | p=0.688 |
| **Birthweight (g), median (IQR)** | 3375  (3100 – 3725) | 3455  (3105 – 3900) | p=0.521 |
| **Birthweight  (INTERGROWTH-21^ST^ percentile), median (IQR)** | 55.29  (27.28 - 82.26) | 55.75  (25.77 - 87.27) | p=0.697 |
| **Birthweight ≥ 4000g, n (%)** | 2 (4.08%) | 10 (20%) | p=0.028 |
| **LGA** (>90percentile****), n (%)** | 10 (20.41%) | 15 (30%) | p=0.643 |
| **SGA*** (<10percentile****), n (%)** | 2 (4.08%) | 4 (8%) | p=0.377 |
| **Neonatal hypoglycaemia,  n (%)** | 4 (8.16%) | 10 (20%) | p=0.148 |

*EAT- Eating Assessment Test **LGA - large for gestational age; ***SGA – small for gestational age

**** Based INTERGROWTH-21^st^ Chart
